# Updates on the Current Treatments for Diabetic Retinopathy and Possibility of Future Oral Therapy

**DOI:** 10.3390/jcm10204666

**Published:** 2021-10-12

**Authors:** Yohei Tomita, Deokho Lee, Kazuo Tsubota, Kazuno Negishi, Toshihide Kurihara

**Affiliations:** 1 Laboratory of Photobiology, Keio University School of Medicine, Tokyo 160-8582, Japan; yohei.tomita@childrens.harvard.edu (Y.T.); deokholee@keio.jp (D.L.); 2 Department of Ophthalmology, Keio University School of Medicine, Tokyo 160-8582, Japan; kazunonegishi@keio.jp; 3 Boston Children’s Hospital, Harvard Medical School, Boston, MA 02115, USA; 4 Tsubota Laboratory, Inc., Tokyo 160-0016, Japan; tsubota@tsubota-lab.com

**Keywords:** diabetic retinopathy, diabetic macula edema, anti-VEGF therapy, vitrectomy, laser photocoagulation, fenofibrate, pemafibrate

## Abstract

Diabetic retinopathy (DR) is a complication of diabetes and one of the leading causes of vision loss worldwide. Despite extensive efforts to reduce visual impairment, the prevalence of DR is still increasing. The initial pathophysiology of DR includes damage to vascular endothelial cells and loss of pericytes. Ensuing hypoxic responses trigger the expression of vascular endothelial growth factor (VEGF) and other pro-angiogenic factors. At present, the most effective treatment for DR and diabetic macular edema (DME) is the control of blood glucose levels. More advanced cases require laser, anti-VEGF therapy, steroid, and vitrectomy. Pan-retinal photocoagulation for non-proliferative diabetic retinopathy (NPDR) is well established and has demonstrated promising outcomes for preventing the progressive stage of DR. Furthermore, the efficacy of laser therapies such as grid and subthreshold diode laser micropulse photocoagulation (SDM) for DME has been reported. Vitrectomy has been performed for vitreous hemorrhage and tractional retinal detachment for patients with PDR. In addition, anti-VEGF treatment has been widely used for DME, and recently its potential to prevent the progression of PDR has been remarked. Even with these treatments, many patients with DR lose their vision and suffer from potential side effects. Thus, we need alternative treatments to address these limitations. In recent years, the relationship between DR, lipid metabolism, and inflammation has been featured. Research in diabetic animal models points to peroxisome proliferator-activated receptor alpha (PPARα) activation in cellular metabolism and inflammation by oral fenofibrate and/or pemafibrate as a promising target for DR. In this paper, we review the status of existing therapies, summarize PPARα activation therapies for DR, and discuss their potentials as promising DR treatments.

## 1. Introduction

Diabetic retinopathy (DR) is a severe complication of diabetes mellitus (DM) and is one of the leading causes of vision loss worldwide. The Vision Loss Expert Group (VLEG) reported that DR accounted for 1.25% of moderate to severe visual impairment and 1.07% of blindness [1]. A meta-analysis reviewed that the percentage of blindness caused by DR varied regionally from 2% in Oceania and East and Southeast Asia to 5.5% in Southern Latin America [2]. They also reported that DR caused blindness in regions with older populations, such as Eastern and Western Europe and Southern Latin America, compared to regions with relatively younger populations [2]. Regarding the type of DM, DR is observed in 42.1% of type 1 DM and 25.5% of type 2 DM [3]. Another study showed that type 1 DM patients were diagnosed with DR in 32.58% of cases, while type 2 DM patients were 23.04% of cases [4]. DR represents a dramatic socioeconomic cost for healthcare systems, and its prevalence has continuously increased in the aging society [5,6,7,8]. The public health burden of DR underlines the significance of searching for promising therapeutic approaches, as well as the advancement of current standards of DR care, including laser, anti-vascular endothelial growth factor (VEGF) therapy, steroid, and vitrectomy [9]. Although surgical or pharmacological therapeutic approaches in DR have been improved, the pathological mechanisms of DR have yet to be fully elucidated. In this study, we review the current proposed pathophysiology of DR and the status of existing surgical and/or pharmacological therapies. Furthermore, we summarize recent promising oral therapies in diabetes; fenofibrate and pemafibrate, well-known agonists of peroxisome proliferator-activated receptor alpha (PPARα) in treatments for dyslipidemia [10] and discuss their recent potentials as promising oral DR treatments.

## 2. Pathophysiology of DR

**Figure 1:**
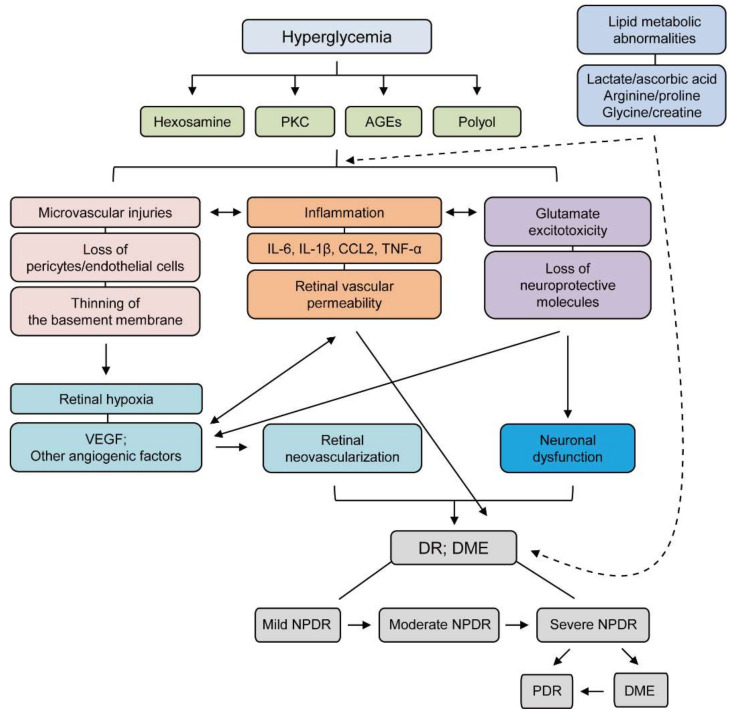
A schematic illustration of the pathophysiology and stages of diabetic retinopathy (DR). Hyperglycemia evokes various pathological metabolic mechanisms such as accumulation of AGEs and induction of PKC, the polyol, and the hexosamine pathways. Microvascular injuries, inflammation, and glutamate excitotoxicity combine to damage the diabetic retina more severely after induction of these pathways. During these processes, representative features are highlighted: loss of pericytes/endothelial cells, thinning of the basement membrane, increases in IL-6, IL-1β, CCL-2, and TNF-α, retinal vascular permeability, and loss of neuroprotective molecules. These outcomes exacerbate neuronal dysfunction, retinal hypoxia, and increases in various angiogenic factors, including VEGF, which ultimately causes retinal neovascularization. All processes are inter-connected to the development and progression of DR. Furthermore, lipid metabolic abnormalities (changes in levels of lactate, ascorbic acid, arginine, proline, glycine, or creatine) in diabetes could aggravate the intensities of retinal injuries. The stages of DR depending on the severity of the disease: mild NPDR (microaneurysm), moderate NPDR (hemorrhage), severe NPDR (more severe hemorrhage, venous beading, and intraretinal microvascular abnormalities), PDR (new vessel formation, retinal detachment, and vitreous hemorrhage); DME (retinal detachment). Solid line; direct interaction, Dash line; indirect interaction. AGEs; advanced glycation end products, PKC; protein kinase C, CCL; Chemokine (C-C motif) ligand TNF; tumor Necrosis Factor, VEGF; vascular endothelial growth factor, DR; diabetic retinopathy, DME; diabetic macular edema, NPDR; non-proliferative diabetic retinopathy.

To date, there have been various insights into the pathophysiology of DR (Figure 1). Firstly, it was simply considered a microvascular disease [11], and hyperglycemia was thought to be the leading cause of retinal microvascular damage [12]. In this regard, pathological metabolic pathways such as accumulation of advanced glycation end products (AGEs) and induction of the protein kinase C (PKC), the polyol, and the hexosamine pathways have been implicated in retinal microvascular damage [13,14].

In the early stage of DR, loss of pericytes can lead to the eventual destruction of the microvasculature, as these cells physiologically enwrap the microvasculature and support endothelial cells [15]. Disturbing the interaction of pericytes with endothelial cells showed aggravation in diabetes-induced microvascular dysfunction [16]. Experimental studies showed that hyperglycemia caused the death of pericytes in vitro and in vivo [17,18]. Additionally, endothelial cell death and thinning of the basement membrane were also described in the early stage of DR [19,20]. Loss of pericytes and endothelial cells can cause capillary occlusion, which leads to retinal ischemic conditions.

Retinal ischemic conditions could lead to more severe stages in DR. Retinal ischemia induces increases in VEGF levels [21,22,23], and VEGF takes part in the mechanism that restores the blood supply to the ischemic retina. This process damages the retina more severely, termed neovascularization-induced retinal damages, and may cause the retina to wrinkle or detach [24,25]. The boundary between proliferative diabetic retinopathy (PDR) and non-PDR (NPDR) is determined by the condition of neovascularization (Figure 1). Neovascularization usually occurs at the vitreoretinal interface [26]. In some cases of NPDR, diabetic macular edema (DME) occurs as a complication. The macula in DME swells with fluid leaked from the damaged vasculature [27]. These conditions from PDR or DME are often related to the development of retinal detachment and vitreous hemorrhage leading to loss of vision in DR patients [27]. Accordingly, research scientists and clinicians have focused on controlling VEGF in DR experimentally and clinically, as they recognized the importance of its principle pathological role [21,23]. Several studies showed that upregulation of VEGF could be mediated by hypoxia-inducible factors (HIFs) [28]. Genes regulated by HIFs (angiogenin-2; ANGIO2, stromal-derived growth factor-1; SDF-1, and platelet-derived growth factor-B; PDGF-B) also play critical roles in retinal neovascularization [29,30]. Therefore, the regulation of HIFs with VEGF and other angiogenic genes has been targeted for the treatment of DR.

Retinal inflammation, as well as retinal degeneration, can be detected from the early stage to the chronic stage in DR. Elevation in various inflammatory cytokines and chemokines (monocyte chemoattractant protein 1/chemokine C-C motif ligand 2; MCP-1/CCL2, tumor necrosis factor-α; TNF-α, interleukin 1β; IL-1β, interleukin 6; IL-6) were described in the serum as well as the vitreous and aqueous humor of patients with DR [31,32,33,34,35,36,37,38,39,40]. Many experimental studies showed that increases in these cytokines and chemokines contributed to cell death of retinal neurons in DR [41,42]. Specifically, massive chronic inflammatory responses (such as infiltration of inflammatory cells to the retina, activation of retinal microglia, and retinal vascular permeability) consisting of numerous cytokines and chemokines are considered critical in the progression of DR, as opposed to individual inflammatory proteins. When it comes to retinal degeneration, increases in the expression of Bax and activation of caspase-3 were detected in retinal neuronal cells in experimental models of diabetes and humans [43,44,45]. Glutamate excitotoxicity with loss of neuroprotective molecules was also suggested to cause retinal neuronal cell death in the diabetic retina [9]. Taken together, there is no doubt that various factors, from microvascular abnormality to retinal cell death, are interconnected in their contributions to the development and progression of DR.

In addition to hyperglycemic dysregulation, lipid metabolic dysregulation has been implicated as a potential risk factor for the development and progression of DR. Clinical studies demonstrated there were strong associations between changes in plasma levels of high-density and low-density lipoproteins (HDL and LDL) and the development of severities of DR as well as diabetes [46,47,48,49,50,51,52]. Abnormal levels of lipids in the blood (termed dyslipidemia), including elevation of LDL, free fatty acids, and triglycerides, reduction of HDL, and inhibition of reverse cholesterol transport gene expressions, could be promoted under insulin-resistant conditions [53,54,55]. A higher frequency of retinal abnormalities was reported in diabetic subjects with dyslipidemia [56,57]. Even though more experimental evidence on lipid metabolic dysregulation with the development and progression of DR are needed, emerging evidence has shown that lipid-modifying drugs could exert possible protective effects in DR at the current stage [58,59].

Recently, several studies performed metabolomics with the vitreous humor in PDR patients. Metabolomics analysis of the vitreous humor could serve as a potential tool to identify new pathways associated with the development and progression of PDR. Barba et al. showed that lactate levels increased while ascorbic acid levels decreased in PDR patients compared to control patients [60]. Paris et al. showed that arginine and proline levels were upregulated in PDR patients [61]. Haines et al. demonstrated that the purine-related pathways were activated, and pyruvate levels increased in PDR patients [62]. Furthermore, our group showed that glycine levels increased, and creatine levels decreased in the vitreous humor of PDR patients [63]. Although research from new angles is underway, the pathophysiology of PDR has not yet been elucidated. More efforts are needed to elucidate the recent issue above.

## 3. Laser Treatment

### 3.1. Treatment for DR

The Diabetic Retinopathy Study (DRS) indicated four risk factors for vision loss in DR. These risk factors include the presence of vitreous or preretinal hemorrhage, the presence of new vessels, the location of new vessels on or near the optic disc, and finally, the severity of pathological conditions in new vessels [64]. According to this study, eyes with three or more risk factors are considered “at high risk” of vision loss in DR.

Laser treatment for DR has been well established for several decades. Since pan-retinal photocoagulation (PRP) can reduce retinal neovascularization, it has been performed to reduce the high-risk development of PDR [65,66]. An analysis using a rabbit model of retinal ischemia showed that photocoagulation suppressed ischemia-induced VEGF, vascular permeability, and angiogenesis promoted by VEGF [67]. In addition, the level of VEGF was lower in eyes treated with PRP compared with that in untreated eyes [68]. For this reason, it is thought that destroying the retinal non-perfusion areas (NPAs) with a laser could reduce pathological angiogenesis from these areas. Therefore, PRP is considered an essential therapeutic tool in controlling DR activity. The optimal timing for PRP is between severe NPDR and early PDR, according to the Early Treatment Diabetic Retinopathy Study (ETDRS) [69]. Clinical trials have shown that PRP reduced the risk of severe vision loss by 50% or more in DR. Furthermore, only 1% of patients and 4% of eyes experienced severe vision loss in 5 years following photocoagulation [70,71]. This approach seemed promising to suppress the progression of DR. However, there were several problems reported in PRP therapies, such as peripheral visual field loss, delayed dark adaptation, and atrophic creep in long-term studies [72,73,74,75].

The pattern scan laser was developed in an attempt to solve these problems [76]. It has been reported that the decrease in the nerve fiber layer (which can be generally seen after PRP) was significantly improved after using the advanced laser. The degree of pain for the patient and the expansion of the coagulation zone were also reduced compared to those in the conventional laser. In addition, it has been reported that the amount of inflammatory cytokines induced following its treatment was lower than that of the conventional methods [77]. Finally, the operation time could be shortened, a significant point during the surgery [78,79]. Despite these benefits, the pattern scan laser has a narrow safety margin because of the short-pulse laser features and is easily influenced by hazy media such as vitreous hemorrhage. It is also known that the coagulation spot tends to shrink over time [80]. Besides, the pattern scan laser has been reported to lead to a higher frequency of retinal neovascularization, iris neovascularization, and neovascular glaucoma than the conventional PRP [81], which implies more investigations into conventional PRP and pattern scan laser therapies in DR may be needed.

Targeted retinal photocoagulation (TRP) has been used for patients with NPDR in some countries. This technique involves selective photocoagulation of the areas in retinal vascular occlusion. It has been reported that the progression rate of PDR could be slowed with selective photocoagulation in patients with multiple NPAs of one papillary diameter or more in NPDR [82]. In addition, a randomized clinical trial showed that extended TRP was effective in early PDR regression with fewer coagulation spots than the conventional PRP [83]. Recently, the navigated pattern laser (NAVILAS), a fundus camera-based photocoagulation system, has been developed. This system enables the delivery of navigated pattern PRP, selectively applied to NPAs [84]. However, in any of the described methods, fluorescence angiography (FA) is necessary to identify NPAs to determine the indication for photocoagulation. On the other hand, a recent randomized trial showed that combination therapy with ranibizumab and widefield FA-guided TRP did not improve visual acuity or reduce the number of anti-VEGF injections compared to ranibizumab alone in DME patients [85]. Therefore, further studies are needed.

### 3.2. Treatment for DME

The ETDRS showed that the focal/grid laser produced better outcomes than the natural course in patients with severe DME [86,87]. Severe macular edema is defined as retinal thickening that involves or threatens the macula’s center. The focal/grid laser is recommended especially for DME that does not include the fovea and does not require frequent visits to the hospital for treatment. However, large or dense coagulation near the macula may result in a paracentral dark spot. Furthermore, complications such as atrophic creep may occur in this chronic condition [88]. Based on these problems, the modified ETDRS laser was proposed in the Diabetic Retinopathy Clinical Research (DRCR) net in 2007 [89]. It is based on direct photocoagulation of capillary aneurysms with a minimally invasive setting and is becoming the standard.

Recently, subthreshold diode laser micropulse photocoagulation (SDM), invisible retinal phototherapy, has been developed to treat DME. It applies heat to the retinal pigment epithelium under conditions that do not cause cell death. The current indications for the SDM for DME are the localized edema outside the fovea and the mild macular edema, including the fovea. To date, several reports have shown a significant efficacy of SDM alone for DME [90,91,92]. Randomized controlled trials have also reported that the SDM is more effective than the modified ETDRS laser [91]. In general, this treatment alone is indicated for cases with relatively mild edema, and combined treatment with anti-VEGF therapy is indicated for severe DME [93].

## 4. Anti-VEGF Treatment

### 4.1. Treatment for DR

Protocol S reported that anti-VEGF treatment (ranibizumab) resulted in significantly better visual acuity than PRP treatment for PDR patients [94]. In addition, the anti-VEGF group had substantially less peripheral visual field loss, faced fewer cases of DME, and a decreased need for vitrectomy compared to those in the PRP group. Other studies have also shown improvements in Diabetic Retinopathy Severity Scale (DRSS) scores as well as a lower risk of vitrectomy and DME with intravitreal anti-VEGF treatment (ranibizumab), compared to PRP [95,96]. The Clinical Efficacy and Mechanistic Evaluation of Aflibercept for Proliferative Diabetic Retinopathy (CLARITY) trial was specifically designed to evaluate the efficacy of PRP versus aflibercept for patients with PDR without DME [97]. A study group showed that ranibizumab suppressed neovascularization and maintained better visual acuity than PRP treatment during the first 12 months of the PRIDE study, but it is not sustained after 24 months under real-world conditions [98,99]. Although these results suggest that anti-VEGF therapy could be more valuable for preventing the progression of DR than PRP, it should be noted that anti-VEGF treatment requires frequent follow-up, compared to general laser photocoagulation, which has a permanent effect on the operated eyes.

With regard to frequent follow-up in anti-VEGF treatment, there is a possibility of worsening retinopathy in patients who stop coming to the clinic. It is reported that anti-VEGF therapy alone has a worse prognosis than photocoagulation if a patient ceases the treatment [100]. Because anti-VEGF drugs are expensive and require multiple visits to the clinic, the management of PDR should be guided by both cost and patient-specific factors such as visit compliance.

### 4.2. Treatment for DME

A multicenter randomized clinical trial showed that anti-VEGF drugs had a therapeutic effect for DME that involves the central macula [101]. The RESTORE study showed that ranibizumab and laser therapy improved visual acuity more than focal/grid laser in patients with DME [102]. The RISE and RIDE study showed that ranibizumab improved visual acuity and macular edema in patients with DME [103]. On the other hand, the VISTA and VIVID studies showed that aflibercept had better visual improvement and more reduction in central retinal thickness than the focal/grid laser alone for DME involving the fovea [104,105]. In terms of the efficacy of bevacizumab, ranibizumab, and aflibercept for DME, it is controversial in randomized clinical trials. Although aflibercept was superior to bevacizumab and ranibizumab in eyes with visual acuity of 20/50 or worse at one year, aflibercept was no longer superior to ranibizumab at two years [106,107]. Another study showed that aflibercept could be more efficient in treating moderate or severe visual acuity loss cases, but aflibercept, bevacizumab, and ranibizumab had comparable effects for mild DME [108]. However, because of the short duration of the drug effect, multiple injections are required to maximize visual improvement. Thus, it is recommended that anti-VEGF therapy for DME involving the fovea may be combined with focal/grid laser therapies to reduce the number of injections [109].

Anti-VEGF injection may increase the risk of high intraocular pressure, infectious endophthalmitis, and cataract [110,111,112]. In addition, the intraocular injection may cause retinal damages or tractional retinal detachment. Furthermore, there is a possibility that anti-VEGF (which is intended to be injected into the vitreous) may unexpectedly diffuse to systemic circulation. The indication for the treatment and re-administration should be determined based on the patient’s general condition as well as their visual acuity and conditions of central retinal thickness.

## 5. Steroid Treatment

### Treatment for DME

Steroid treatment is indicated when edema is diffuse throughout the macula. Steroids have an anti-inflammatory effect that helps to downregulate both pro-inflammatory and pro-angiogenic mediators, which are crucial for the development of DME. General steroid treatments have the possibility of multiple side effects, so topical corticosteroid treatments are preferable for DME. There are several options for administering steroids, such as intravitreal injection, subtenone injection, and dexamethasone intravitreal implant (DEX).

Intravitreal triamcinolone acetonide injection (IVTA) has played an essential role in treating DME for many years, especially prior to the approval of anti-VEGF injections [113,114]. Substantial improvements in macular thickness and visual acuity have been reported with IVTA [115]. Another group showed that a single IVTA injection to DME induced a significant improvement in macular thickness and visual acuity at three months after the treatment [116]. However, because of the short effective duration, multiple injections are required to maintain its efficacy. A clinical trial reported that there was no significant change between the bevacizumab vs. bevacizumab + IVTA groups in terms of best corrected visual acuity and central macular thickness changes, compared to the baseline at 24 weeks. Nonetheless, the bevacizumab + IVTA group showed earlier visual improvement [117]. Thus, combined therapy with anti-VEGF treatment may be recommended for the early visual improvement.

Subtenone triamcinolone acetonide injection (STTA) is also used to treat DME patients. Several studies have shown that STTA reduced retinal thickness and improved visual acuity [118,119]. However, it is still controversial regarding results for the DME treatment [118,119]. In addition, because it also has a short duration of effects, patients need multiple injections as they do with IVTA. To overcome this problem, STTA is often used in conjunction with anti-VEGF treatment. For example, a retrospective study compared the combination therapy of STTA and intravitreal anti-VEGF injection with anti-VEGF monotherapy to treat anti-VEGF-resistant DME. The thirty-eight eyes treated with the combination therapy showed significantly improved visual acuity and macular thickness after six months of treatment. Although STTA injection is less effective for short-term DME than IVTA injection, it has been shown that STTA resulted in less steroid-responsive intraocular pressure elevation than intravitreal steroids [120,121]. Another group showed that STTA treatment with bevacizumab improved morphological changes and reduced the frequency of bevacizumab treatments in DME patients [122]. Thus, STTA may be a useful adjunctive therapy in anti-VEGF-resistant DME in the case of existing concerns for steroid-induced glaucoma.

The DEX implant, an FDA-approved DME treatment, has become an alternative injection method to IVTA and STTA in several countries. It has provided a longer-term option for steroid therapy. Several retrospective studies have shown that in anti-VEGF-resistant eyes, a single DEX implant improved macular thickness and visual acuity [123,124,125,126]. Another group treated 16 eyes with a single DEX implant and reported significant improvements in macular thickness at one, two, and three months. However, the DRCR network showed that combination therapy of DEX implant and anti-VEGF did not improve visual acuity at twenty-four weeks more than anti-VEGF treatment alone [127]. Intravitreal fluocinolone acetonide is another implant with longer effective periods than the DEX implant. Multiple studies have shown that the intravitreal fluocinolone acetonide implant could be useful for treating anti-VEGF-resistant DME, with sustained beneficial visual acuity and macular thickness outcomes [128,129,130,131,132]. The systematic review included seven randomized clinical trials that suggested IVTA and surgical implantation of steroids may improve visual outcomes in eyes with refractory DME [133]. However, since each treatment can carry additional glaucoma and cataract progression risks, special care is needed to avoid these complications [134,135]. In addition, ophthalmologists need to be cautious of noninfectious endophthalmitis (NIE) in that ocular inflammation occurs after intravitreal injection of corticosteroids [136]. A recent study indicates that the incidence rate of NIE lies between 0.1% and 7.3% [137].

## 6. Surgical Treatment

### 6.1. Treatment for DR

PDR patients with dense and recurrent vitreous hemorrhage or tractional retinal detachment close to the retina or rhegmatogenous retinal detachment require surgical treatments. Vitrectomy refers to the surgery for retinal and vitreous diseases. During the surgery, surgeons remove the vitreous and replace it with another solution. Photocoagulation is needed to attach the retina if there is an NPA or retinal tear. In PDR patients, the longer a macular detachment occurs, the more difficult it becomes to restore vision due to the deterioration of macular photoreceptor function. In recent years, the development of small-incision vitrectomy such as the 23-, 25-, and 27-gauge and wide-angle viewing systems (for instance, Resight^®^) has contributed to the improvement of vitreoretinal surgery [138,139,140,141]. These techniques make vitrectomy less invasive and safer so that earlier vitrectomy, in cases with macula dragging due to fibrovascular tissue overlying the macula, may be indicated in patients with PDR [142]. Furthermore, these operations are shown to be safer following anti-VEGF pretreatment [143,144]. Thus, the indications of vitrectomy for the treatment of PDR have been expanded by the early intervention [145].

However, there are many cases of poor prognosis in PDR following vitrectomy. Permanent vision loss may occur due to retinal detachment involved in the macula, glaucoma, or ischemic changes. Re-bleeding may occur after vitrectomy for vitreous hemorrhage, although a small incision vitrectomy reduces this recurrence [146,147]. Cataract formation should be considered after vitrectomy in patients with phakia. Although the incidence is low, it is also necessary to pay attention to postoperative endophthalmitis.

### 6.2. Treatment for DME

Vitrectomy has been reported to improve visual acuity for DME accompanied by an epiretinal membrane or thickened posterior vitreous cortex with vitreomacular traction [148,149,150]. Vitreoretinal surgery may also be considered a treatment option when the outcomes were not satisfactory from retinal photocoagulation and drug therapies such as anti-VEGF and steroids. Posterior vitreous detachment and peeling of the internal limiting membrane can be performed for DME. This is because the inner limiting membrane could become a scaffold for proliferative myofibroblasts [151], which may cause the recurrence of macular edema with the epiretinal membrane.

Long-term results showed improved visual acuity after vitrectomy for DME in 496 eyes, comparable to those shown in anti-VEGF therapy [152]. Vitrectomy caused a more significant central macular thickness reduction than IVTA 12 months after treatment, although it showed no statistically significant change in visual acuity [153]. It was reported that vitrectomy was morphologically effective after six months compared to the grid laser, but there was no significant difference in visual function. In addition, there was no significant difference in morphology or visual function after one year. On the other hand, it has been reported that the presence or absence of an internal limiting membrane detachment did not affect the prognosis of visual function in vitrectomy for DME without macular traction [154]. Recently, Imai et al. reported the effectiveness of cystotomy for long-term anatomical and functional improvements of refractory cystoid macular edema secondary to DR. However, it is necessary to accumulate many cases for the concrete conclusion of the effectiveness of cystotomy [155,156]. Overall, there is the possibility of the same risks as other vitrectomies, and care must be taken when choosing potential patients.

Importantly, all of these treatments are for advanced diseases. There is no safe and effective ophthalmic treatment other than controlling of blood glucose levels, even if the early stage of DR is observed. Thus, we need preventive treatments for DR.

## 7. Fenofibrate Therapy in DR

Fenofibrate is a well-known peroxisome proliferator-activated receptor alpha (PPARα) agonist. PPARα is one of the members in the nuclear receptor family of ligand-activated transcription factors [13]. Heterodimerization of PPARα with the retinoic X receptor regulates the transcription of genes involved in cellular metabolism [13]. Fenofibrate could reduce free fatty acids levels by upregulating the synthesis of molecules for fatty acid transport and β-oxidation through the activation of PPARα [13,157,158]. Furthermore, fenofibrate has the potential to induce an increase in the synthesis of apolipoproteins and high-density lipoprotein cholesterol [159,160,161]. In this regard, fenofibrate could be used as a therapeutic drug in metabolic syndrome. Fenofibrate has been effectively used in the US to manage patients with dyslipidemia since 1998 [162]. As metabolic syndrome is a common finding in patients with diabetes, the future use of fenofibrate in the management of patients with DR was also examined in the Fenofibrate Intervention and Event Lowering in Diabetes (FIELD) study [163] and the Action to Control Cardiovascular Risk in Diabetes (ACCORD)-Eye study [164]. Based on the FIELD study, the fenofibrate-administered group showed a significant reduction of relative risk in need for laser treatment for DME and PDR. When it comes to the ACCORD-Eye study, the progression of DR was significantly reduced in the fenofibrate-statin-administered group compared to that in the only statin-administered group. Taken together, fenofibrate was suggested as a promising therapeutic for slowing the progression of DR.

Experimental animal studies also supported the notion that fenofibrate may have protection against DR [165,166,167]. Oral administration of fenofibric acid, the active metabolite of fenofibrate, reduced ganglion cell death and preserved amplitudes in oscillatory potentials and implicit time of b-wave in diabetic db/db mice [165]. Upregulated expressions of *Il-6*, *Il-1β*, *P53*, *Bax*, and *Vegf* and their protein expressions in the diabetic rat retina were reduced by oral administration of fenofibrate [166]. Similar effects of fenofibrate treatment were found in human retinal microvascular endothelial cells under high glucose stimulation [166]. Another study showed that oral administration of fenofibrate attenuated oxidative stress and neuroinflammation in the diabetic mouse retina via increasing expressions of a master regulator of antioxidative defense, nuclear factor erythroid 2–related factor 2 (Nrf2) and its target genes, including heme oxygenase 1 (Ho-1), and decreasing the formation of reactive oxygen species [167]. Taken together, fenofibrate appears viable for the treatment of DR.

Even though fenofibrate is a generally well-tolerated drug, its effects on increases in serum levels of creatinine were continuously reported [168,169,170]. This finding raised concerns regarding deleterious damages to renal function. Safety issues surrounding the use of fenofibrate are still debated. As such, fenofibrate is not highly recommended for use in patients with severe renal impairment. Along with this issue, researchers have been attempting to develop better therapeutics for PPARα activation.

## 8. Pemafibrate Therapy in DR

Pemafibrate is a new selective PPARα modulator, recently synthesized by Kowa Company, Ltd. as a more efficient and safer alternative to fenofibrate. Clinical studies in Japan demonstrated that pemafibrate showed superior effects on cellular metabolism compared to fenofibrate by improving liver function and increasing serum creatinine levels less likely or decreasing the estimated glomerular filtration rate [10,171,172]. One of the possible reasons is that pemafibrate is metabolized in the liver and excreted into the bile, while other fibrates, including fenofibrate, are predominantly excreted from the kidney. In this regard, pemafibrate could be a safer option in patients with severe renal impairment.

Experimental animal studies showed that pemafibrate could be used as a promising drug in diabetes and DR. In diabetic mice, oral administration of pemafibrate reduced plasma levels of triglycerides and vasoconstrictive eicosanoids [173]. Furthermore, impaired endothelial function in diabetic mice was attenuated by treatment with pemafibrate [173]. In high-fat diet mice with a femoral artery endothelial denudation injury, oral administration of pemafibrate increased serum levels of fibroblast growth factor 21 (FGF21) and decreased serum levels of insulin, attenuating neointima formation [174]. In our previous paper, long-term oral administration of pemafibrate improved blood glucose levels, modulated cellular metabolism, upregulated PPARα target genes in the liver (not in the retina), and increased serum levels of FGF21, to protect against diabetes-induced retinal dysfunction (amplitudes in oscillatory potentials) in mice [175]. Furthermore, we found that FGF21 could increase retinal protein expression of synaptophysin, one of the important molecules for maintaining oscillatory potentials [175]. Another study suggested that oral administration of pemafibrate directly inhibited retinal inflammation in diabetic rats by decreasing expressions of MCP-1 and VCAM-1 [176]. Furthermore, oral administration of pemafibrate inhibited diabetes-induced retinal vascular leukostasis by upregulating thrombomodulin expression [176]. Pemafibrate was recently suggested to protect against *N*-methyl-*D*-aspartate (NMDA) excitotoxicity-induced cell death in the rat retina (analyzed by TUNEL assay) [177]. This effect was associated with the inhibition of phosphorylated c-Jun, one of the possible links to the expression of apoptosis-related genes [177]. In terms of retinal neovascularization in DR, we previously demonstrated that oral administration of pemafibrate showed a significant reduction in retinal neovascularization in a murine model of oxygen-induced retinopathy [178]. Furthermore, a significant increase in serum levels of FGF21 and decreases in retinal HIF-1α immunostaining and *Vegfa* expression were found after oral administration of pemafibrate [178]. Taken together, pemafibrate therapy experimentally shows promise as a potential therapy for diabetes and DR.

Pemafibrate to Reduce cardiovascular OutcoMes by reducing triglycerides IN patiENts with diabeTes (PROMINENT), a phase 3 randomized clinical trial, has been ongoing (ClinicalTrials.gov Identifier: NCT03071692). Patients with dyslipidemia with type 2 diabetes were recruited in this study. Although DR had also been evaluated in the sub-analysis, because the number of recruited subjects for the DR study did not meet the criteria, it was suspended. However, if pemafibrate has therapeutic effects for metabolic syndromes, drug-repositioning of pemafibrate could be applied to treat retinal diseases, including DR, in the future.

## 9. Conclusions

**Figure 2:**
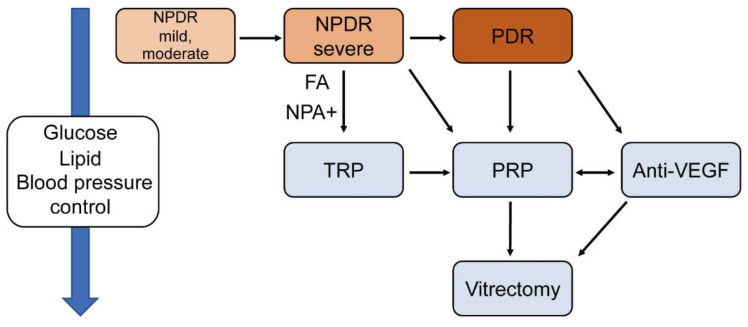
A flowchart of suggested treatment for diabetic retinopathy (DR) without macular edema. DR is categorized as non-PDR (NPDR) and PDR. NPDR falls into three subcategories: mild, moderate, and severe. The control of glucose and lipid levels and blood pressure is crucial at any stage. NPDR; non-proliferative diabetic retinopathy, DME; diabetic macular edema, TRP; Targeted retinal photocoagulation, FA; Fluorescein angiography, NPA; non-perfusion area.

**Figure 3:**
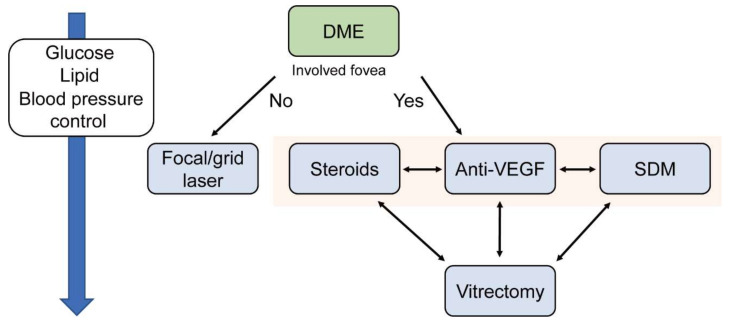
A flowchart of suggested treatment for diabetic macular edema (DME). The strategy of treatment is decided by whether the edema includes the fovea or not. Controlling levels of glucose, lipid, and blood pressure is crucial at any stage. SDM; subthreshold diode laser micropulse photocoagulation, DME; diabetic macular edema.

This study summarized current therapies such as laser, anti-VEGF, steroid, and surgery for DR and DME. Figure 2 and Figure 3 show suggested treatments for PDR and DME. Typically, patients with severe NPDR can be treated with PRP, however, if the NPA can be assessed in FA, then TRP can be a viable option as well. Patients with PDR need PRP or anti-VEGF therapy depending on the clinical and socioeconomic situation of the patients (Figure 2). The treatment of DME is divided according to whether the edema involves the fovea. If the edema does not include the fovea, patients could be treated by focal/ grid laser. If it includes the fovea, patients may be treated by anti-VEGF or steroid therapy. SDM is also an option, if available (Figure 3).

Through every stage, the control of glucose and lipid levels is essential. However, all of these treatments can be applied for progressed diseases. Even if the early stage of DR is observed, clinicians can only contribute control of blood glucose levels, lipids, and hypertension [163,164,179,180,181]. It is also reported that controlling diabetic nephropathy, anemia, and sleep apnea is important for preventing DME [182]. This systemic medication is crucial to prevent and slow down the progression of DR. In addition, existing treatments are for vascular disorders, not for neurological disorders. Therefore, neuroprotection or preventive therapy for the early stage of DR needs to be developed as soon as possible. Future treatments may also be required with fewer side effects than the current therapies. At this point, many clinical studies with various therapeutic strategies are ongoing for slowing the progression of DR (searched in ClinicalTrials.gov).

**Figure 4:**
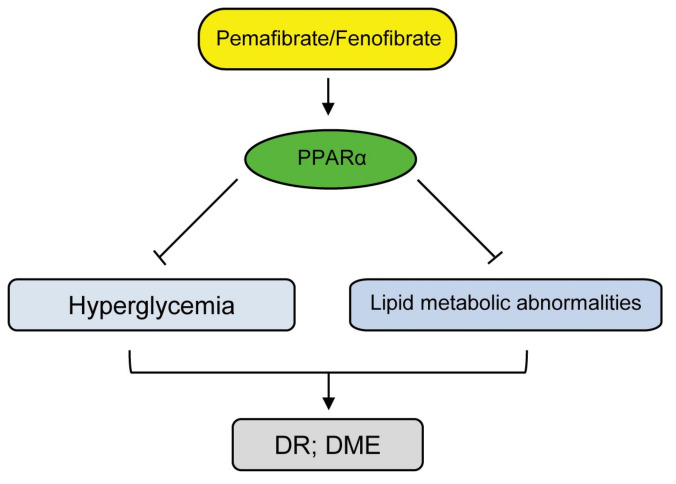
A flowchart of promising treatments for diabetic retinopathy (DR) and diabetic macular edema (DME). PPARα activation by pemafibrate/fenofibrate is associated with modulating energy homeostasis in hyperglycemia and lipid metabolic abnormalities through the regulation of both lipid and glucose levels. Continuous control of levels of glucose and lipid is available with this promising treatment. DR; diabetic retinopathy, DME; diabetic macular edema.

Based on the outcomes summarized in this review, oral non-invasive PPARα agonists (fenofibrate and pemafibrate) treatments could be one of the promising therapeutics to prevent the development and/or the progression of DR (Figure 4). Even though more investigations are needed, we hope that ophthalmologists can prevent DR with these drugs.
